# An inhibitor of the Keap1-Nrf2 protein-protein interaction protects NCM460 colonic cells and alleviates experimental colitis

**DOI:** 10.1038/srep26585

**Published:** 2016-05-24

**Authors:** Meng-Chen Lu, Jian-Ai Ji, Yong-Lin Jiang, Zhi-Yun Chen, Zhen-Wei Yuan, Qi-Dong You, Zheng-Yu Jiang

**Affiliations:** 1 Jiang Su Key Laboratory of Drug Design and Optimization, China Pharmaceutical University, Nanjing 210009, China; 2 Department of Medicinal Chemistry, School of Pharmacy, China Pharmaceutical University, Nanjing 210009, China

## Abstract

Ulcerative colitis (UC) is a chronic relapsing-remitting form of inflammatory bowel disease (IBD) that increases the risk of colorectal cancer, the third most common malignancy in humans. Oxidative stress is a risk factor for the development of UC. The Keap1-Nrf2-ARE pathway is one of the most important defensive mechanisms against oxidative and/or electrophilic stresses. In this study, we identified **CPUY192018** as a potent small-molecule inhibitor of the Keap1-Nrf2 PPI, investigated the cyto-protective effects of **CPUY192018** on the NCM460 colonic cells and evaluated whether treatment with the inhibitor of the Keap1-Nrf2 PPI exerts protection on an established experimental model of UC induced by dextran sodium sulfate (DSS). Our study clearly demonstrated that **CPUY192018** had a cytoprotective effect against DSS in both NCM460 cells and mouse colon via the activation of Nrf2 signaling. These results suggested that activation of Nrf2 by directly inhibiting the Keap1-Nrf2 PPI may be beneficial as a treatment for UC.

Ulcerative colitis (UC) and Crohn’s disease (CD) are the two major clinically defined inflammatory bowel diseases (IBD). Historically, IBD is more common in Western countries than in other regions. However, in recent years, the incidence and prevalence of IBD have increased with time in other regions, and it has now emerged as a global disease and has drawn increasing research interest^1^. The detailed etiology of UC is still obscure, but it is now widely accepted that it is a chronic relapsing-remitting inflammatory condition that may affect the entire colon. Notably, UC increases the risk of colorectal cancer, which is the third most common malignancy in humans. In contrast to CD, which can affect any part of the gastrointestinal tract, the pathology of UC is restricted to the colonic mucosa^2^, and the depth of the inflammation in UC is proximal to the epithelium. Thus, colonocytes are implicated in the pathogenesis of this disease, and the dysfunction of the intestinal epithelium is the main cause of UC. The single layer of columnar intestinal epithelial cells constitute a huge surface area of approximately 100 m^2 ^3. In addition to the obvious function as the physical barrier, the intestinal epithelium has various biological functions, including processing and absorbing dietary nutrients and maintaining the intestinal homeostasis^4^. Damage to the intestinal epithelial cells can disrupt the barrier function of the intestinal epithelium, facilitating an aberrant immune response and inflammatory conditions. Thus, the intact intestinal epithelium is critical for the healthy gut, and cyto-protective agents that could target the intestinal epithelial cells would be beneficial for the treatment of UC. However, management of UC has so far relied on nonspecific immunosuppressive therapies (such as steroids), antibiotics, and biologicals, most of which have targeted the proinflammatory tumor necrosis factor (TNF) pathway^5^.

NF-E2-related factor 2 (Nrf2) is a cyto-protective transcription factor that can up-regulate certain detoxification and antioxidant genes. These genes all contain the cis-acting regulatory element ARE (antioxidant responsive element) in their promoter sequences, and can be positively regulated by the ARE sequence. Nrf2 binds to ARE, induces the transcription of genes and serves as a key node of the ARE-driven cellular defense system. The genes transcriptionally regulated by the Nrf2-ARE signaling pathway encode detoxification enzymes and antioxidant proteins that play important roles in the cellular defense system, especially in oxidative stress modulation. Numerous studies have proven that Nrf2 protects many cell types and organ systems from a broad spectrum of toxic insults and disease pathogenic processes^6^,^7^.

Nrf2 activity is regulated mainly by Kelch-like ECH-associated protein-1 (Keap1). Under basal conditions, Keap1 negatively regulates the Nrf2 activity by mediating the polyubiquitination of the Nrf2 protein. Keap1 is a substrate adaptor component in the Cullin3 (Cul3)-based ubiquitin E3 ligase complex, which recognizes Nrf2 by protein-protein interaction (PPI) and shepherds Nrf2 towards polyubiquitination and degradation by the 26S proteasome^8^,^9^,^10^. Keap1-mediated negative regulation of Nrf2 prevents the unnecessary activation of Nrf2 under normal conditions. Under stress conditions, the excess oxidative and/or electrophilic agents can covalently modify the cysteine residues in the Keap1 protein, which induces a mutation-like effect on this reside of the Keap1 protein and cause a conformational change of the complex^11^,^12^. The altered conformation of the complex inhibits the ubiquitination process and allows newly synthesized Nrf2 to avoid Keap1-meditated suppression. Subsequently, Nrf2 is imported into the nucleus, where it induces the transcription of downstream genes by binding to ARE. The Nrf2-regulated cell defense system is activated to counteract oxidative and other environmental stressors.

The protective effects of Nrf2 on the colon have also been validated. Nrf2 plays an important role in protecting the intestinal integrity through regulation of proinflammatory cytokines and induction of phase II detoxifying enzymes^13^. Nrf2-deficient mice are more sensitive to dextran sulfate sodium (DSS)-induced colitis, and the increased severity was found to be associated with the down-regulation of detoxification enzymes and antioxidant proteins^13^. DSS treatment resulted in increased lipid peroxidation and severe oxidative damage in the colons of the Nrf2-deficient mice^14^. The severe oxidative damages caused by the lack of Nrf2 is consistent with the key role for Nrf2 in protection against intense oxidative stress. Oxidative stress has been considered a potential driving force in the induction and progression of UC^15^. There is substantial evidence that ROS play a key role in the pathogenesis of colitis^16^,^17^. Excess levels of ROS may damage both nuclear and mitochondrial DNA, RNA, lipids and proteins by nitration, oxidation and halogenation reactions, leading to an increased mutation load, impaired regulation of cellular growth and even cell death. Nrf2 controls multiple antioxidant systems in the colon epithelium including the GSH-based antioxidant system, the Phase I drug oxidation, reduction and hydrolysis system and the Phase II drug conjugation system^18^. The sophisticated defense systems in the colonic mucosa are involved in maintaining the redox homeostasis and protecting the cells against ROS and electrophilic agents. Thus, Nrf2 activation would be an alternative strategy to exert therapeutic effects on colitis. Some Nrf2 activators have been proven to be beneficial for the treatment of colitis. For example, sulforaphane pre-treatment can mitigate DSS-induced acute colitis^19^.

Recently, Keap1-Nrf2 PPI inhibitors have been developed as a novel class of Nrf2 activators^20^,^21^,^22^. This type of agent can rescue Nrf2 from Keap1-directed ubiquitination by competitively and directly disrupting the Keap1-Nrf2 PPI. Unlike the traditional Nrf2 activators, the Keap1-Nrf2 inhibitors interact with Keap1 by non-covalent interactions, and there are no electrophilic groups in their chemical structures. This molecular mechanism of action can improve the selectivity of the agents and may be beneficial for further applications. Previously, we successfully identified the first nanomole Keap1-Nrf2 inhibitor, **CPUY192002**^23^. We subsequently optimized the solubility of the inhibitor using medicinal chemistry methods, resulting in a more potent Keap1-Nrf2 PPI inhibitor, **CPUY192018**^24^. There has recently been considerable progress in the development of a potent Keap1-Nrf2 PPI inhibitor. In this study, we investigated the cyto-protective effects of **CPUY192018** on the NCM460 colonic cells and evaluated whether treatment with a Keap1-Nrf2 PPI inhibitor exerts protection on an established experimental model of UC induced by DSS.

## Results

### Biophysical characterization of potent Keap1-Nrf2 PPI inhibitors

**Figure 1:**
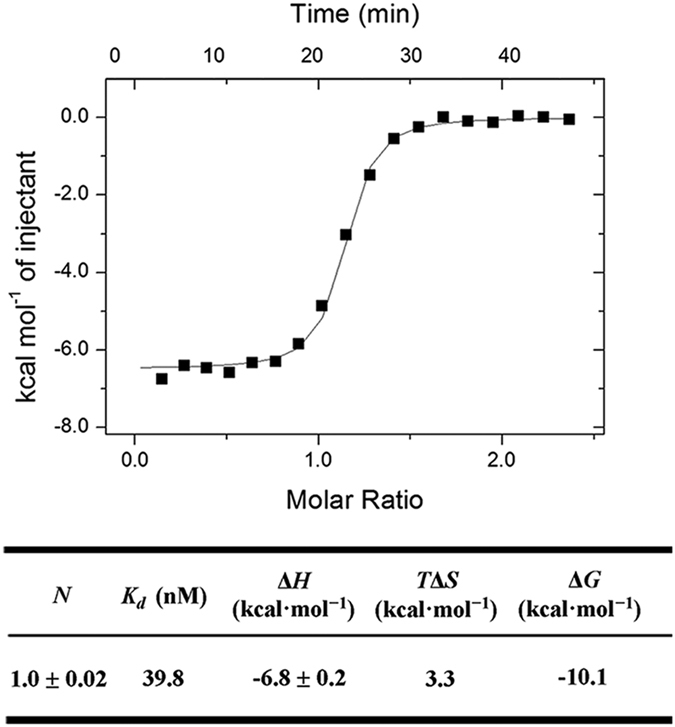
ITC profile of the titration of the Keap1 Kelch domain with CPUY192018. The thermodynamic parameters of the interaction of Keap1 with **CPUY192018** as determined by the ITC Assay are listed in the table. *N* is the stoichiometric coefficient. *K*_*d*_ is the binding constant. Δ*H*, Δ*S*, and Δ*G* refer to the changes in binding enthalpy, entropy, and total Gibbs free energy, respectively. Δ*G* is calculated according to the equation Δ*G* = Δ*H* − *T*Δ*S*, where *T* is the absolute temperature used for the ITC experiment.

Previously, we have identified the binding determinants of the Keap1-Nrf2 PPI^25^ and reported the identification of the nanomolar inhibitor **CPUY192018**^24^. **CPUY192018** can mimic the binding pattern of Nrf2, especially the multiple polar interactions in the P1 and P2 sub-pockets of the Keap1 cavity. This inhibitor has a strong binding affinity for Keap1, with an IC_50_ of 14.4 nM in a fluorescence polarization assay as previously reported 24. In this study, an isothermal titration calorimetry (ITC) assay, which is commonly employed to assess the thermodynamics and affinity of a ligand-receptor interaction^26^, was applied to quantify the ligand binding. The resulting ITC profile showed a typically reversible 1:1 binding stoichiometry, suggesting that one small molecule bound to one molecule of Keap1. The curve-fitting analysis parameters indicated **CPUY192018** has a *K*_*d*_ value of 39.8 nM. The thermodynamic analysis showed that the binding of **CPUY192018 **has a strong enthalpic component (ΔH = −6.8 ± 0.2 kcal·mol^−1^) and a favorable entropy (TΔS = 3.3 kcal·mol^−1^), which suggested a synergistic enthalpy/entropy-driven process in the binding of **CPUY192018** to Keap1 (Fig. 1).

### CPUY192018 activated the Nrf2-ARE pathway *in vitro*

**Figure 2:**
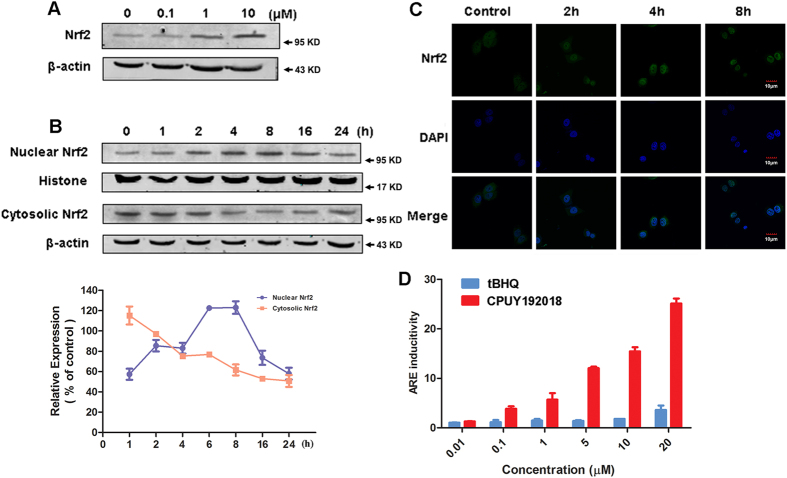
CPUY192018 activated the Nrf2-ARE pathway *in vitro*. **(A)** Effect of **CPUY192018** on the induction of the Nrf2 protein expression. **(B)** Effect of **CPUY192018** on the nuclear translocation of the Nrf2 protein. At various time poits after the treatment with **CPUY192018** (10 μM), nuclear and cytoplasmic cell extracts were prepared from the NCM460 cells and subjected to western blot analysis. Histone and β-actin served as markers for nuclear and cytosolic Nrf2 proteins, respectively. Densitometric analysis was performed to determine the relative ratios of the protein in each fraction. The data were normalized to the β-actin expression and are expressed as the means + SEM of three individual experiments. The data were analyzed using Image J 1.44p. **(C)** Immunofluorescence staining of Nrf2 at the indicated times in the NCM460 cells treated with 10 μM **CPUY192018**. Nrf2 and the nuclei were labeled with FITC and DAPI, respectively. The bars indicate the magnification (10 μm). **(D)** ARE induction by **CPUY192018** and t-BHQ in the HepG2−ARE−C8 cells. The cells were exposed to the compounds or DMSO for 12 h. The activities are shown as the ratio to the DMSO control. The values shown are the means ± SEM (n = 3 independent observations).

Keap1 is the key regulator of Nrf2 and utilizes a unique cyclic mechanism to target Nrf2 for ubiquitination and proteasomal degradation through the Keap1-Nrf2 PPI^11^,^12^. Disrupting the Keap1-Nrf2 PPI can inhibit the Keap1-mediated negative regulation of Nrf2 and elevate the Nrf2 protein level. Thus, we first investigated the effect of **CPUY192018** on the Nrf2 protein level. As shown in Fig. 2A, **CPUY192018** increased the level of the Nrf2 protein in the NCM460 colonic cells in a concentration- and time-dependent manner. Translocation of Nrf2 into the nucleus is essential for the transactivation of ARE-regulated genes. Therefore, to further investigate the effects of **CPUY192018** on the Nrf2-ARE activation, we examined the subcellular localization of the Nrf2 and determined both the nuclear and cytoplasmic Nrf2 levels after treatment of the NCM460 cells with **CPUY192018**. Immunoblotting analysis of the cytosolic and nuclear Nrf2 fractions (Fig. 2B) as well as the immunofluorescence analyses (Fig. 2C) showed Nrf2 accumulation in the nuclei of the NCM460 cells. The Nrf2 nuclear localization was initiated within 2 h, maximally increased at 8 h, and subsequently declined after 16 h of treatment with 10 μM **CPUY192018**.

To further explore the biological relevance of the **CPUY192018**-induced activation of Nrf2-ARE signaling, we evaluated the relative potency of **CPUY192018** in comparison to the classical Nrf2 activator t-BHQ using the ARE-luciferase reporter assay. HepG2-ARE-C8 cells that had been stably transfected with a luciferase reporter were treated with different concentrations (0.01, 0.1, 1, 5, 10 and 20 μM) of **CPUY192018** or t-BHQ for the indicated times. Parallel cell viability assays indicated no apparent cytotoxicity of the treatment of **CPUY192018**. Significantly, as shown in Fig. 2D, **CPUY192018** (at nanomolar to sub-micromolar concentrations) was more potent in inducing the Nrf2-ARE pathway than t-BHQ, which was effective only in the micromolar range. Moreover, **CPUY192018** also increased the induction of ARE in a clearly concentration-dependent manner.

### CPUY192018 stimulated the transcription of the Nrf2-ARE regulated cytoprotective genes in NCM460 cells in an Nrf2-dependent manner

It has been confirmed that increased ARE transactivation caused by the accumulation of Nrf2 in the nucleus can subsequently augment the expression of numerous ARE-responsive cytoprotective and antioxidant enzymes including heme oxygenase-1 (HO-1), glutamate-cysteine ligase (GCLM) and glutathione peroxidase 2 (GPx2)^27^. HO-1 is the rate-limiting enzyme in heme catabolism and is induced by a wide variety of oxidative agents. Nrf2 translocates into the nucleus and positively regulates HO-1 transcription, which may therefore function as an endogenous defensive mechanism to attenuate inflammatory events in the gastrointestinal tract^28^. GCLM is a modulatory subunit of glutamate cysteine ligase (GCL), the rate-limiting enzyme for glutathione (GSH) synthesis^29^. GPx2 is predominantly expressed in the intestinal epithelium^30^, and its expression is precisely regulated by Nrf2^31^. Studies of GPx2 suggest that this enzyme may play an important role in the maintenance of mucosal homeostasis^19^.

**Figure 3:**
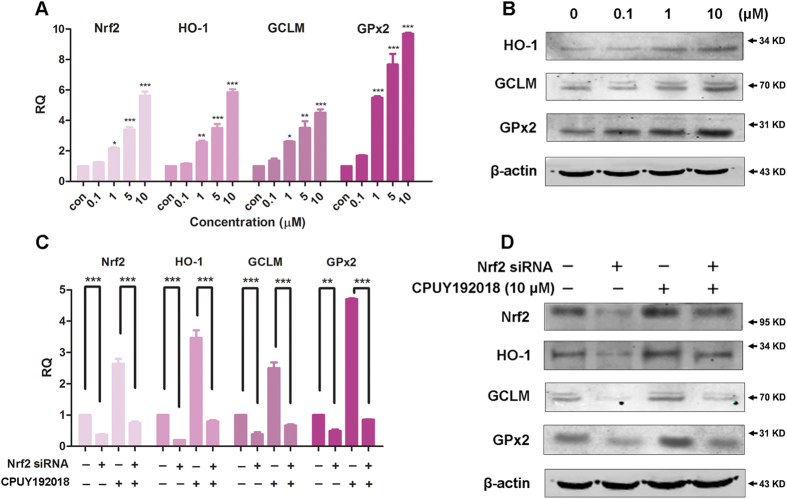
CPUY192018 stimulated the transcription of the Nrf2-ARE-regulated cytoprotective genes in the NCM460 cells in an Nrf2-dependent manner. **(A)** Quantitative real-time PCR analysis of Nrf2, HO-1, GCLM, and GPx2 in the NCM460 cells. The mRNA levels of Nrf2 and the Nrf2-targeted genes were measured at 10 h after treatment of the NCM460 cells with various concentrations (0.1, 1, 5, 10 μM) of **CPUY192018**. β-actin was used to normalize the expression of these genes. **(B)** Western blot analysis of the Nrf2 downstream proteins HO-1, GCLM, and GPx2 in the NCM460 cells after treatment with various concentrations (0, 0.1, 1, 10 μM) of **CPUY192018** for 8 h. **(C)** The mRNA expression of Nrf2 and the Nrf2-regulated genes after exposure to Nrf2 siRNA and **CPUY192018**. The NCM460 cells were treated with Nrf2 siRNA (80 nM), **CPUY192018** (10 μM), or Nrf2 siRNA (80 nM) plus **CPUY192018** (10 μM). Additional NCM460 cells were treated with a scrambled duplex for use as the blank control. The expression of the Nrf2, HO-1, GCLM and GPx2 genes was quantified using qRT-PCR. **(D)** Western blot analysis of Nrf2 and the Nrf2-regulated proteins after exposure to Nrf2 siRNA and **CPUY192018**. The NCM460 cells were treated with Nrf2 siRNA (80 nM), **CPUY192018** (10 μM), or Nrf2 siRNA (80 nM) plus **CPUY192018** (10 μM). Additional NCM460 cells were treated with DMSO for use as the blank control. The values shown are the means ± SEM (n = 3 independent observations). ***P < 0.001, **P < 0.01, and *P < 0.05, one-way ANOVA with Tukey–Kramer posttest.

To ascertain the effects of **CPUY192018** on the expression of the Nrf2-ARE-driven genes in NCM460 cells, the mRNA levels of Nrf2 and the three selected genes described above, HO-1, GCLM, and GPx2, were examined. The quantitative real-time PCR (qRT-PCR) analysis showed that exposure of the NCM460 cells to 0.1–10 μM **CPUY192018** for 10 h strongly increased the transcription of Nrf2 and the Nrf2-regulated genes in a concentration-dependent manner. At the highest concentration of **CPUY192018** (10 μM), the levels of the mRNA for Nrf2, HO-1, GCLM, and GPx2 increased 5.6-, 5.8-, 4.5- and 9.7-folds, respectively, compared to the DMSO control (Fig. 3A). Consistent with these results, the evaluation of the Nrf2-targeted proteins by western blotting demonstrated that the treatment of the NCM460 cells with 0.1–10 μM **CPUY192018** induced HO-1, GCLM, and GPx2 protein expression in a concentration-dependent manner (Fig. 3B).

To define the role of Nrf2 in the **CPUY192018**-induced antioxidant gene expression, the NCM460 cells were transfected with an siRNA against human Nrf2. Consistent with the silencing of Nrf2, markedly reduced protein and mRNA levels of Nrf2 together with its target genes were observed in the Nrf2 siRNA-treated NCM460 cells (Fig. 3C,D). As expected, knocking down Nrf2 remarkably decreased the **CPUY192018**-induced up-regulation of Nrf2 and its target genes HO-1, GCLM, and GPx2. These results proved that **CPUY192018** activates Nrf2-ARE signaling in an Nrf2-dependent fashion.

### CPUY192018 produced cytoprotective effects against the DSS-induced injury in the NCM460 cells

A growing body of evidence suggests that oxidative stress is a major pathogenic factor for the development of chronic inflammatory diseases such as UC^15^,^32^,^33^. Oxidative injury might be a potential driving force in the progression of UC and carcinogenesis. The present study therefore aimed to elucidate whether **CPUY192018**-induced antioxidant activities can actually protect against oxidative injury. Dextran sodium sulfate (DSS) has been reported to induce oxidative injury in NCM460 cells^34^. This chemical is a sulfated polysaccharide that has been widely used to induce inflammation in animal models of IBD^34^.

**Figure 4:**
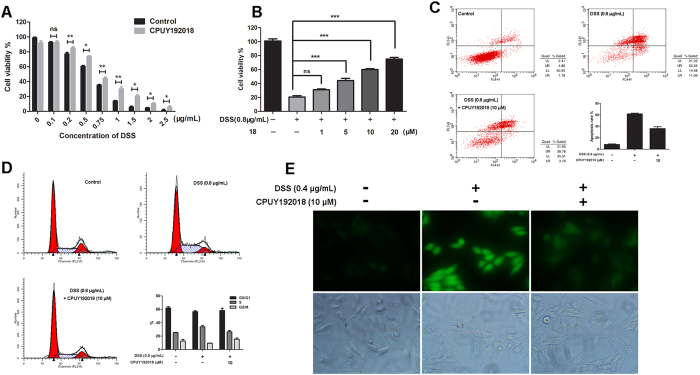
Effects of **CPUY192018** on DSS-induced cell injury in the NCM460 cells. **(A)** Protective effects of **CPUY192018** against the DSS-induced cell damage. The NCM460 cells were pretreated with 10 μM **CPUY192018** for 10 h then exposed to various concentrations of DSS for an additional 12 h. The cell viability was determined using the MTT assay. **(B)** Dose-dependent protective effects of **CPUY192018** against the DSS-induced cell damage. The NCM460 cells were pretreated with 1–20 μM **CPUY192018** for 10 h then exposed to 0.8 μg/mL DSS for an additional 12 h. The cell viability was determined using the MTT assay. **(C)** Flow cytometric analysis of the apoptotic rate. The NCM460 cells were treated with 10 μM **CPUY192018** for 10 h before being exposed to 0.8 μg/mL DSS for an additional 8 h. The cells were stained with FITC-Annexin V-PI, and the apoptotic rates were detected by flow cytometry. The statistical analysis of the apoptotic rates is shown in the figure. **(D)** The effect of **CPUY192018** on the cell cycle in the NCM460 cells. The NCM460 cells were treated with 10 μM **CPUY192018** for 10 h before being exposed to 0.8 μg/mL DSS for an additional 8 h. At the end of this treatment, the cells were trypsinized, incubated with RNase, stained with propidium iodide (PI), and analyzed by flow cytometry. The statistical analysis of the ratio of the NCM460 cells in the G0/G1, S and G2/M phases of the cell cycle is shown in the figure. **(E)** Living Cell Microscopy. The NCM460 cells were pretreated with 10 μM **CPUY192018** for 10 h and then exposed to 0.4 μg/mL DSS for an additional 6 h. After this treatment, the NCM460 cells were stained with 10 μM cH_2_DCF-DA for 20 min at 37 °C and living cell fluorescence microscopy was performed. The values shown are the means ± SEM (n = 3 independent observations). *p < 0.05, **p < 0.01, ***p < 0.001, one-way ANOVA with Tukey–Kramer posttest.

We first used the MTT assay to evaluate the protective effects of **CPUY192018** against the DSS-induced cell damage. As shown in Fig. 4A, treatment with DSS (average MW 36,000–50,000, MP Biomedicals, LLC) caused a decrease in cell viability. In particular, when the concentration of DSS was higher than 0.75 μg/mL, the survival rate of NCM460 cells was less than 50%. **CPUY192018** antagonized the cell damage caused by DSS, and this protective effect was concentration-dependent (Fig. 4B). We further evaluated the ability of **CPUY192018** to protect against DSS-induced apoptosis in NCM460 cells using flow cytometry. As shown in Fig. 4C, exposure to 0.8 μg/mL DSS caused the apoptosis rate to increase to approximately 64% in the NCM460 cells. Pretreatment with 10 μM **CPUY192018** significantly decreased the DSS-induced apoptosis level. Then, we investigated the effect of **CPUY192018** on the cell cycle in the NCM460 cells. The data in Fig. 4D show that DSS induced an S-phase cell-cycle block in the NCM460 cells, and the arrest was restored to the normal level by **CPUY192018**.

Previous studies have illustrated that DSS can significantly increase the intracellular ROS level^34^. To investigate the effect of **CPUY192018** on the DSS-induced increases in the ROS levels in the NCM460 cells, the fluorogenic cellular ROS indicator c-H_2_DCF-DA was used to stain the cells. As shown in Fig. 4E, the cells exposed to 0.4 μg/mL DSS for 6 h showed a significantly stronger living cell fluorescence microscopic signal than that of control sample due to the higher oxidation of the incorporated c-H_2_DCF-DA. **CPUY192018** could remarkably suppress the cellular c-H_2_DCF-DA staining, which indicates its protective effect against the oxidative stress in the NCM460 cells.

These results proved that **CPUY192018** exerted cytoprotective effects against the DSS-induced oxidative injury in the NCM460 cells. Pretreatment with **CPUY192018** significantly increased the survival rate of the NCM460 cells, conferred protection against the DSS-induced cell apoptosis and inhibited the DSS-induced S-phase cell-cycle block in the NCM460 cells. These cytoprotective effects of **CPUY192018** may be due in part to the diminished intracellular ROS levels induced by DSS exposure.

### CPUY192018 alleviated DSS-induced chronic ulcerative colitis

**Figure 5:**
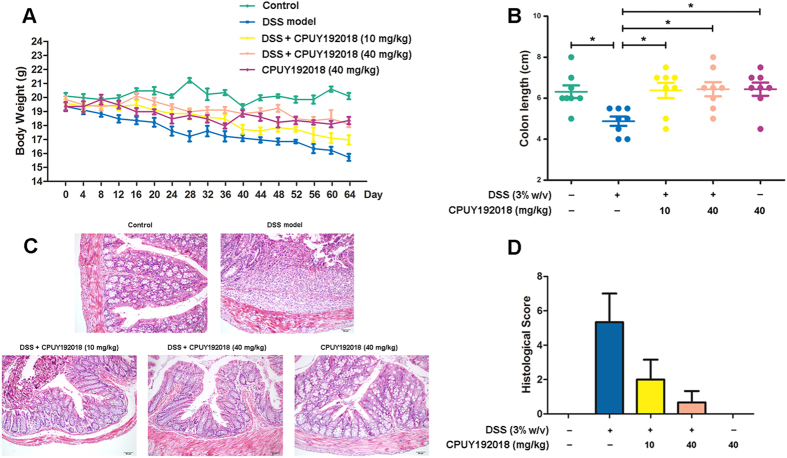
**CPUY192018** ameliorated the pathological symptoms in the DSS-induced mouse model of UC. The animals were randomly assigned to one of the five treatment groups: Control group (the mice received regular drinking water); DSS model group (the mice received 3% w/v DSS in drinking water); DSS + **CPUY192018** (10 mg/kg) group (the mice received 3% w/v DSS in drinking water together with administration by gavage of 10 mg/kg of **CPUY192018**); DSS + **CPUY192018** (40 mg/kg) group (the mice received 3% w/v DSS in drinking water together with administration by gavage of 40 mg/kg of **CPUY192018**); and **CPUY192018** (40 mg/kg) group (the mice received administration by gavage of 40 mg/kg of **CPUY192018**). **(A)** Gradual changes in body weight during the DSS administration in mice. **(B)** The comparison of the colon length on day 64. **(C)** Representative histological images of distal colon sections stained with hematoxylin and eosin (H&E). Magnifications×100. **(D)** Histologic inflammatory score. The results are expressed as the means + SEM (n = 8, in each group). *p < 0.05, **p < 0.01 and ***p < 0.001, one-way ANOVA with Tukey–Kramer posttest.

Considering the impact of Nrf2 in UC, we hypothesized that **CPUY192018** would have a therapeutic potential and tested the effect of **CPUY192018** on Nrf2 activation *in vivo* using a DSS-induced mouse model of UC. After treatment with regular drinking water for 2 days for adaptation, female C57BL/6 mice (6–8 weeks of age, weighing 18–20 g) were randomized (8 animals in each group) to receive four cycles of DSS (3% w/v). Each cycle consisted of 8 days of DSS in drinking water followed by 8 days of water without DSS. In this model, the mice given DSS (3% w/v) in their drinking water developed serious symptoms of chronic colitis including decreased body weight, diarrhea, liquid stools, rectal bleeding and shortened colon lengths compared to the mice that received regular drinking water. Oral administration of **CPUY192018** ameliorated the progressive chronic injury as indicated by attenuation of the body weight loss and the severity of diarrhea and rectal bleeding during the DSS treatment and also reduced the DSS-induced shortening of the colon length. Histological analysis of the dysplasia in the colonic crypts was also performed using hematoxylin and eosin (H&E) staining. The animals exposed to the DSS (3% w/v) exhibited typical inflammatory changes including disorganized architecture of colonic mucosa, crypt damage, infiltration of inflammatory cells into the mucosal tissue and expansion of lamina propria relative to the control group. In the mice treated with **CPUY192018**, the pathogenic conditions were improved as indicated by a well-preserved colonic structure, elimination of the inflammatory cells, and prevention of the lamina propria expansion (Fig. 5C). Likewise, the mice treated with the DSS exhibited high histological disease scores including high-grade dysplasia and colonic adenoma, and these injuries were alleviated by the **CPUY192018** administration as reflected by the low histological disease scores (Fig. 5D). The viability of mice was not affected by the administration of **CPUY192018** (40 mg/kg/day) for 64 days. Instead, mice treated with **CPUY192018** alone exhibited far fewer infiltrating cells, a remarkably lower grade of mucosal injury and less edema.

### CPUY192018 relieved the DSS-induced inflammatory conditions in the colon

**Figure 6:**
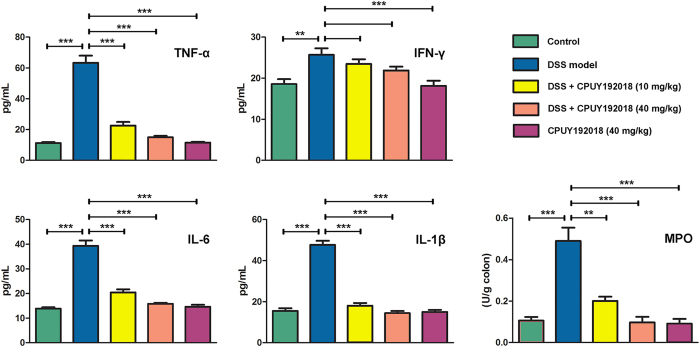
Quantification of the inflammatory cytokines TNF-α, IFN-γ, IL-6, and IL-1β and the MPO activity in the colon homogenates from the C57BL/6 female mice. The results are expressed as the means + SEM. *p < 0.05, **p < 0.01 and ***p < 0.001, one-way ANOVA with Tukey–Kramer posttest.

During the progression of IBD, a complex array of inflammatory signaling processes impairs the intestinal epithelial function and leads to the recruitment of inflammatory cells to the site of injury. We therefore further assessed several inflammatory markers to evaluate the effects of **CPUY192018** on the DSS-induced colonic inflammatory conditions. After four cycles of DSS administration, we examined the levels of TNF-α, IFN-γ, IL-6 and IL-1β using ELISAs and measured the MPO activity in the colon tissue. In response to oxidative stress, pro-inflammatory cytokines are often overproduced and they, in turn, can also cause oxidative stress in the target cells. Several pro-inflammatory cytokines including TNF-α, IL-1β and IL-6 are overproduced when the redox-sensitive nuclear factor-B (NF-κB) is activated by oxidative stress. As shown in Fig. 6, all of the inflammatory indicators increased markedly in the DSS-treated mice compared with the control group. Both low-dose and high-dose **CPUY192018** treatments significantly decreased the expression of the inflammatory cytokines TNF-α, IL-6 and IL-1β compared to the mice given DSS alone. In the case of MPO, the mice given DSS alone showed an obvious increase in the MPO activity compared to the control group. The **CPUY192018** treatment clearly reduced the MPO activity to a level similar to that of the control group. **CPUY192018** at a dose of 40 mg/kg/day had no effect on the pro-inflammatory factors or the MPO activity. Based on these results, **CPUY192018** appears to modulate the activation and migration of the inflammatory cells, thereby reducing the cytokine production in the colon. A marked reduction was observed in the IL-6, TNF-α and IL-1β levels, which was accompanied by a decrease in the MPO activity. This resulted in an improvement in the inflammatory symptoms.

### CPUY192018 produced cytoprotective effects and reduced the oxidative damage by activating Nrf2 and up-regulating Nrf2-targeted cytoprotective proteins

**Figure 7:**
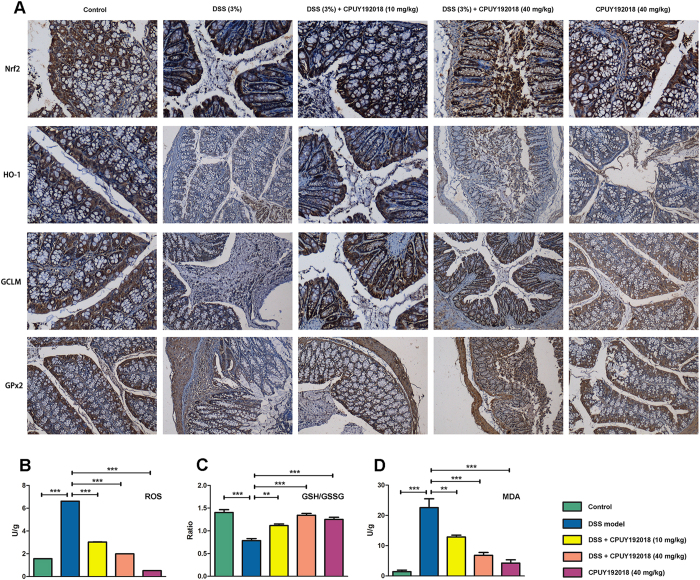
CPUY192018 attenuated the DSS-induced oxidative stress and apoptosis in the mouse colons. **(A)** Immunohistochemical detection of Nrf2 together with its target proteins HO-1, GCLM and GPx2 levels in the mouse colons. Magnifications ×200. The ROS level **(B)** the ratio of GSH/GSSG **(C)** and MDA level **(D)** in the colons were measured. The results are expressed as the means + SEM. *p < 0.05, **p < 0.01 and ***p < 0.001, one-way ANOVA with Tukey–Kramer posttest.

Because we have confirmed that **CPUY192018** protected against the DSS-induced cell damage in the NCM460 colonic cells by activating the expression of Nrf2 downstream cytoprotective proteins, we then investigated whether **CPUY192018** could exert similar protective effects in the colon of the DSS-induced mouse UC model. As shown in Fig. 7A, administration of **CPUY192018** clearly up-regulated the expression levels of Nrf2 together with its target proteins HO-1, GCLM and GPx2 in the mouse colon, which further confirmed the *in vivo* Nrf2-ARE activation effects of **CPUY192018**.

The DSS-induced generation of ROS contributes substantially to the inflammatory as well as the oxidative tissue injury, which play important roles in UC^35^. Here, we first examined whether enhancing the Keap1-Nrf2-ARE signaling by **CPUY192018** affected the ROS level that was elevated by DSS. We found that **CPUY192018** administration significantly reduced the ROS level (Fig. 7B). Keap1-Nrf2-ARE also controls the GSH-based antioxidant system, which is involved in protecting the colon. Therefore, we further measured the GSH/GSSG ratio in the colon as an indicator of the GSH-based antioxidant system. The ratio was decreased in the DSS-treated mice, but treatment with **CPUY192018** increased this ratio significantly (Fig. 7C). Collectively, the increased GSH/GSSG ratio combined with the elevation of Gpx2, which is responsible for the majority of the glutathione-dependent hydrogen peroxide-reducing activity in the epithelium of the gastrointestinal tract, confirmed the enhanced antioxidant capacity. Then, we further evaluated the oxidative stress in the colon, and the reactive species malondialdehyde (MDA) was chosen as a marker for oxidative stress. We found that administration of **CPUY192018** significantly reduced the MDA level of DSS model group but did not affect the level of the normal group (Fig. 7D), which indicates that **CPUY192018** can relieve the oxidative stress in the DSS-induced mouse model of UC.

## Discussion

Starting with a structure-based design aimed at identifying small-molecule inhibitors of the Keap1-Nrf2 PPI followed by a medicinal chemical optimization approach, we identified **CPUY192018** as a potent small-molecule inhibitor for the modulation of the Keap1-Nrf2 PPI. The strong binding affinity of **CPUY192018** to the Keap1 protein was further confirmed by the ITC assay. On the basis of the extraordinary Keap1-Nrf2 PPI inhibition potency, **CPUY192018** was used as a small-molecule probe to investigate the Keap1-Nrf2-ARE pathway. First, we confirmed that **CPUY192018** could increase the protein level of Nrf2 in the NCM460 colonic cells in a concentration- and time-dependent manner. The increased Nrf2 protein then translocated into the nucleus and subsequently induced the expression of the ARE-driven genes including HO-1, GCLM and GPx2. Silencing Nrf2 sharply reduced the induction of Nrf2 and its target genes HO-1, GCLM, and GPx2 by **CPUY192018**, indicating that **CPUY192018** elevated the expression of the Nrf2 downstream genes in an Nrf2-ARE-dependent manner.

Nrf2-ARE signaling regulates the constitutive and inducible transcription of various genes that encode detoxification enzymes and antioxidant proteins, which have pivotal roles in the defense against cellular oxidative stress. Our results demonstrated that the induction of Nrf2-regulated cytoprotective proteins by **CPUY192018** could confer protection against DSS-induced oxidative injury in NCM460 cells. The cell viability experiments showed that the **CPUY192018** treatment significantly reduced the DSS-induced cell death in the NCM460 colonic cells. Pretreatment with **CPUY192018** also protected the NCM460 cells against the DSS-induced apoptosis and suppressed the DSS-induced cell-cycle block. Using c-H_2_DCF-DA fluorescence as an indicator, we also observed that **CPUY192018** markedly decreased the DSS-induced cellular ROS generation. On the basis of these results, we further evaluated the *in vivo* protective effects of **CPUY192018** using a DSS-induced mouse model of UC. The mice given **CPUY192018** exhibited a significant improvement in the DSS-induced severe pathogenic conditions as indicated by the pathological indices. With regard to the colitis severity, further assessment on the antioxidant activity and ROS generation in the colonic tissue also confirmed the antioxidative activity of **CPUY192018** in the *in vivo* UC model. **CPUY192018** markedly attenuated the DSS-induced colonic inflammatory conditions as indicated by the reduced levels of inflammatory markers including TNF-α, IL-6 and IL-1β and the MPO activity in the colon tissue. The immunohistochemical analysis demonstrated that administration of **CPUY192018** effectively elevated Nrf2 as well as its target proteins HO-1, GCLM and GPx2 in the mouse colon, which confirmed the *in vivo* Nrf2-ARE activation effects of **CPUY192018**. The reduced ROS level along with the increased GSH/GSSG ratio further confirmed the enhanced antioxidant capacity. Finally, the MDA level was assessed as a biomarker of the lipid oxidation. The decreased levels of lipid and protein oxidation supported the cytoprotective effects of an active Nrf2 signaling pathway against the proinflammatory stimulus of the DSS exposure.

Our study clearly demonstrated that **CPUY192018**, a potent small-molecule inhibitor of Keap1-Nrf2 PPI, has a cytoprotective effect against the DSS-induced damage in both the NCM460 cells and the mouse colon, and this effect is due to the activation of Nrf2 signaling. Ulcerative colitis affects the colonic mucosa, which is continuously exposed to a diverse mixture of chemicals and oxidative stressors^4^. Therefore, the impairment of intestinal epithelium is a primary contributor to the pathogenesis of UC. Oxidative stress can result in an accumulation of oxidative DNA damage in the inflamed tissue, finally leading to cellular dysfunction or death^15^. Cells possess endogenous defense systems including the Nrf2-ARE signaling pathway that counteract cellular oxidative stress and attenuate oxidative injury^3^. Many *in vivo* studies have demonstrated that Nrf2 is a key regulator of the cellular response to oxidative stress and is crucial in mitigating inflammation in multiple experimental models^6^,^14^,^36^,^37^. Activating Nrf2-ARE signaling by small-molecule Keap1-Nrf2 PPI inhibitors can enhance the endogenous defense system of cells to counteract oxidative stress. The increased cytoprotective proteins responsive to Nrf2 activation may protect the intestinal epithelial cells against oxidative stress and high levels of ROS. This activity supports the integrity of the intestinal epithelium, which is the physical and biochemical barrier to the external environment, and finally alleviates the UC pathogenesis^3^. Endogenous antioxidants such as glutathione (GSH) can restore cellular homeostasis by effectively inhibiting the increase in the ROS level^6^. Expression of HO-1 was also induced in the epithelial cells^38^. The three by-products of HO-1, biliverdin, free iron and carbon monoxide (CO), together contribute to the protection of cells against proinflammatory factors and oxidative stress in intestinal disease models^28^. In addition, Nrf2 coordinately regulates a wide array of detoxification, antioxidant, and NADPH-regenerating enzymes, which orchestrate the adaptive responses to diverse forms of stress^18^. A large number of inflammation–associated signals including nuclear factor kappa B (NF-κB)^39^ are susceptible to changes in the intracellular reduction-oxidation state^40^. Diminishing the cellular oxidative stress by up-regulating the Nrf2 downstream antioxidative proteins may thus result in the inhibition of the NF-κB signaling, which could provide a possible anti-inflammatory mechanism for the decrease of DSS-induced cytokines. Moreover, activation of Nrf2 could attenuate the cellular injury and inhibit the release of the cell contents that may trigger or contribute to the expression of the pathological inflammatory responses. This process could therefore improve the cellular microenvironment and subsequently activate a set of signaling pathways responsive to inflammation. In the present work, **CPUY192018** reduced the levels of the inflammatory cytokines and produced a cytoprotective effect in the DSS-induced model of UC by significantly increasing the expression of the Nrf2-regulated cytoprotective proteins. These results suggest that activating Nrf2 by directly inhibiting the Keap1-Nrf2 pathway may be beneficial for the treatment of UC.

It has been verified that dietary supplementation with widely used antioxidants such as vitamin E^41^, carotenes and other antioxidative agents^42^ can actually increase the risk of cancer and accelerate the progression of tumors^42^,^43^. Cells are equipped with endogenous defense mechanisms that counteract the intrinsic and extrinsic stresses, and activating the body’s own antioxidant defense system may be a better choice. Prompt activation of Nrf2 plays a pivotal role in maintaining the cellular redox balance. Compared with other Nrf2 activation methods, targeting the Keap1-Nrf2 PPI to enhance the Nrf2 activity showed some attractive characteristics. Most traditional Nrf2 activators are electrophilic agents, and the molecular mechanism of action of these agents is to covalently modify the cysteine residues in the Keap1 protein. However, cysteine residues are ubiquitous in cells. For this reason, the electrophilic Nrf2 activators may have some off-target effects that could compromise their further development. Thus, competitively and reversibly disrupting the Keap1-Nrf2 PPI may possess advantages with respect to selectivity and safety. Therefore, we believe that Keap1-Nrf2 PPI inhibition is a promising strategy for activating the Keap1-Nrf2-ARE pathway-mediated defense system and may be beneficial in the treatment of UC. A better understanding of the Nrf2 signaling and the role of the Keap1-Nrf2 PPI in it may provide new therapeutic approaches for UC, and could also enable the development of potential therapies to attenuate or treat diseases in which oxidative stress is involved.

## Methods

### ITC Assay

The ITC assay was carried out as previously described and can be found in the supporting information.

### Cell Culture Conditions

HepG2 cells stably transfected with a luciferase reporter (HepG2-ARE-C8) were kindly provided by Professor Dr. A. N. Tony Kong (Rutgers University, Piscataway, NJ) and Prof. Rong Hu (China Pharmaceutical University, Nanjing). Cells were maintained in modified RPMI-1640 medium (GiBco, Invitrogen Corp., USA) with 10% fetal bovine serum (FBS) (GiBco, Invitrogen Corp., USA) and penicillin/streptomycin in a 37 °C incubator with 5% CO_2_. Human NCM460 colonocytes (INCELL, San Antonio, TX) were cultured in Dulbecco’s Modified Eagle Medium (Life Technology^TM^, 1645798) supplemented with 10% (v/v) FBS and penicillin/streptomycin.

### ARE-Luciferase Activity Assay

The experimental procedures were carried out as reported previously^24^. Generally, HepG2-ARE-C8 cells were plated in 96-well plates at a density of 4 ×104 cells/well and incubated overnight. The cells were exposed with different concentrations of test compounds, with tBHQ serving as positive control, DMSO as a negative control, and the luciferase cell culture lysis reagent as a blank. After 12 h of treatment, the medium was removed and 100 μL of cold PBS was added into each well. Then the cells were harvested in the luciferase cell culture lysis reagent. After centrifugation, 20 μL of the supernatant was used for determining the luciferase activity according to the protocol provided by the manufacturer (Promega, Madison, WI). The luciferase activity was measured by a Luminoskan Ascent (Thermo Scientific, USA). The data were obtained in triplicates and expressed as fold induction over control.

### Immunofluorescence

NCM460 cells were treated with CPUY192018 (10 μM) at indicated times, then incubated at 4 °C overnight with Nrf2 primary antibodies (abcam, UK). After washing with PBS, cells were incubated at 37 °C for 1 h with FITC-labeled secondary goat anti-rabbit IgG antibody (Life Technology). Cells were then stained with fluorochrome dye DAPI (Santa Cruz Biotechnology, Santa Cruz, CA) to visualize the nuclei and observed under a laser scanning confocal microscope (Olympus Fluoview FV1000, Japan) with a peak excitation wave length of 570 nm and 340 nm.

### RNA Extraction and qRT-PCR Analysis

The experimental procedure of quantitative real-time RT-PCR was previously reported. Total RNA of NCM460 cells was extracted from the treated cells using TRIzol reagent (Invitrogen). Then the RNA was converted to cDNA by reverse transcriptase (PrimeScript RT reagent kit) according to the manufacturer’s instructions. Quantitative real-time RT-PCR analysis of Nrf2, HO-1, GCLM and Gpx2 were performed by using the StepOne System Fast Real Time PCR system (Applied Biosystems). The values are expressed as the fold of the control. All genes’ mRNA expression was normalized against β-Actin expression. Primers used for qRT-PCR are listed as follows: Nrf2 (Sense primer: AACCACCCTGAAAGCACGC, Antisense primer: TGAAATGCCGGAGTCAGAATC); HO-1 (Sense primer: ATGGCCTCCCTGTACCACATC, Antisense primer: TGTTGCGCTCAATCTCCTCCT); GCLM (Sense primer: TTGGAGTTGCACAGCTGGATTC, Antisense primer: TGGTTTTACCTGTGCCCACTG); GPx2 (Sense primer: GTGCTGATTGAGAATGTGGC, Antisense primer: AGGATGCTCGTTCTGCCA).

### Western blot analysis

Anti-Gpx2 (sc-54604) and anti-GCLM (sc-22755) antibodies were purchased from Santa Cruz Biotechnology (Santa Cruz, CA, USA). Anti-β-action (AP0060) and anti-Nrf2 (BS1258) were purchased from Bioworld (Bioworld, USA). Anti-HO-1 (#5853S) were bought from Cell Signaling Technology (USA). Isolation of cell fractions and Western blotting were performed as detailed previously^44^. Briefly, the extracts were separated by SDS-PAGE and then electrotransferred to PVDF membranes (Perkin Elmer, Northwalk, CT, USA). Membranes were blocked with 1% BSA for 1 h followed by incubation with a primary antibody at 4 °C overnight. Then they were washed and treated with a DyLight 800 labeled secondary antibody at 37 °C for 2 h. The membranes were screened through the odyssey infrared imaging System (LI-COR, Lincoln, Nebraska, USA).

### Transfection of Small Interfering RNA (siRNA)

Predesigned siRNA against human Nrf2 (catalogue no. 115762) and control scrambled siRNA (catalogue no. 4611) were purchased from Biomics (Biomics, China). NCM460 cells were plated at a density of 6 × 10^5 ^cells per 60 mm dish. Cells were transfected with 80 nM siRNA against Nrf2 or 80 nM scrambled duplex using Lipofectamine 2000 (Invitrogen). After 24 h incubation, fresh medium was added, and the cells were cultured for another 48 h. The cells were then treated with compounds for an additional 6 h and lysed for use in qRT-PCR.

### Flow cytometric detection of apoptosis

NCM460 cells in logarithmic phase in a six well tissue culture plate were treated with test samples for indicated time. Then they were harvested, washed and resuspended with PBS. Apoptotic cells were determined with an FITC Annexin V Apoptosis Detection Kit (Beyotime, China) according to the manufacturer’s protocol. Briefly, the cells were washed and subsequently incubated for 15 min at room temperature in the dark in 100 μL of 1× binding buffer containing 5 μL of Annexin V-FITC and 5 μL of PI. Apoptosis was analyzed by FAC Scan laser flow cytometer (Guava easycyteHT, Millipore, CA).

### Cell cycle analysis

The ratio of NCM460 cells in the G0/G1, S and G2/M phases of cell cycle was determined by their DNA content. NCM460 cells were treated with 10 μM CPUY192018 for 10 hours before exposed to 0.8 μg/mL DSS for an additional 8 h. Cells were then harvested, washed twice with cold PBS, and fixed with 75% ice-cold ethanol overnight. Fixed cells were washed twice with cold PBS and incubated with 5 μl of 100 μg/ml RNase A for 30 min at 37 °C. After incubation, the cells were stained with 50 μg/ml PI for 30 min in the dark and analyzed by flow cytometry. Untreated cells were used as a control.

### Living Cell Microscopy

NCM460 cells were seeded in 6-well plates at the density of 70–80% confluence per well for overnight incubation. Then the cells were treated with test samples for indicated time. After treatment, cells were washed once with 2 ml of 10% PBS and stained with 10 μM cH2DCF-DA (S0033, Reactive Oxygen Species Assay Kit, Beyotime, China) in the dark at 37 °C for 20 min in DMEM medium free with FBS. Analysis was done with a fluorescence microscope (OLYMPUS DP72, Japan) equipped with a U-RFL-T power supply.

### Animal experiments

#### Animals

Animal studies were conducted according to protocols approved by Institutional Animal Care and Use Committee of China Pharmaceutical University. All animals were appropriately used in a scientifically valid and ethical manner. Female C57BL/6 mice (Comparative Medicine Centre, Yangzhou University, China), 6−8 weeks of age weighing 18−20 g, were acclimatized under a 12 h light/dark cycle at 22 °C and 60% humidity for 2 days before the experiments and fed with a standard laboratory rodent diet and water. Mice were given free access to diet and water during the course of experiments.

#### Induction of Chronic DSS Colitis and Treatment

Mice were randomized (8 animals in each group) to receive four cycles of DSS (3% w/v). DSS (molecular weight of 36,000–50,000) was obtained from MP Biomedicals, LLC. Each cycle consisted of 8 days of DSS in drinking water followed by 8 days of water without DSS. The animals were assigned randomly to one of four treatment groups: Control group (mice received regular drinking water), DSS model group (mice received 3% w/v DSS in drinking water), DSS + CPUY192018 (10 mg/kg) group (mice received 3% w/v DSS in drinking water together with administration by gavage of 10 mg/kg of CPUY192018), DSS + CPUY192018 (40 mg/kg) group (mice received 3% w/v DSS in drinking water together with administration by gavage of 40 mg/kg of CPUY192018), CPUY192018 (40 mg/kg) group (mice received administration by gavage of 40 mg/kg of CPUY192018). Body weight was measured every 4 days and the animals were sacrificed at day 64 by cervical dislocation for macroscopical inspection and their colons were removed.

#### IL-1β, IL-6, TNF-α and IFN-γ Production

Levels of IL-6 (IL-6 (m) ELISA kit, Nanjing Senbeijia Biological Technology Co., LTD), TNF-α (TNFα(m) ELISA kit, Nanjing Senbeijia Biological Technology Co., LTD), IFN-γ (IFNγ(m) ELISA kit, Nanjing Senbeijia Biological Technology Co., LTD) and IL-1β (IL-1β(m) ELISA kit, Nanjing Senbeijia Biological Technology Co., LTD) were evaluated in the colon homogenate using commercially available kits according to the manufacturer’s instructions.

#### Measurement of MDA, MPO, GSH/GSSG ration and ROS

MDA (A003-1, Malondialdehyde (MDA) assay kit (TBA method), Nanjing Jiancheng Bioengineering Institute, China), MPO activity (A044, Myeloperoxidase assay kit, Nanjing Jiancheng Bioengineering Institute, China), the ratio of GSH/GSSG (GSH and GSSG Assay Kit, S0053, Beyotime, China) and ROS level (S0033, Reactive Oxygen Species Assay Kit, Beyotime, China) were evaluated in the colon homogenate using commercially available kits according to the manufacturer’s instructions.

#### Histopathological examination

Specimens of the colon fixed with 10% buffered formalin were embedded in paraffin. Each section (4 μm) was stained with H&E. The fixed sections were examined by light microscopy for the presence of lesions. Histological evaluation of the severity of inflammation was performed using a scoring system^45^, by a pathologist who was blinded to the treatment.

#### Immunohistochemical analysis

The dissected colon tissues were prepared for immunohistochemical (IHC) analysis of the expression patterns of Nrf2, HO-1, GCLM, and GPx-2. Four-lm sections of 10% formalin-fixed, paraffin-embedded tissues were cut on silanized glass slides and deparaffinized three times with xylene and rehydrated through graded alcohol bath. The deparaffinized sections were heated by using microwave and boiled twice for 6 min in 10 mM citrate buffer (pH 6.0) for antigen retrieval. To diminish nonspecific staining, each section was treated with 3% hydrogen peroxide and 4% peptone casein blocking solution for 15 min. For the detection of respective protein expression, slides were incubated with Nrf2 (Bioworlde, USA), HO-1 (Cell Signaling Technology, USA), GCLM (Santa Cruz Biotechnology), and GPx-2 (Santa Cruz Biotechnology) antibodies at room temperature for 40 min in Tris-buffered saline containing 0.05% Tween 20, and then developed using respective horseradish peroxidase (HRP)-conjugated secondary antibodies (rabbit, mouse, or goat) EnVisionTM System (Dako). The peroxidasebinding sites were detected by staining with 3,3′-diaminobenzidine tetrahydrochloride (Dako). Finally, counterstaining was performed using Mayer’s hematoxylin.

## Additional Information

**How to cite this article**: Lu, M.-C. *et al*. An inhibitor of the Keap1-Nrf2 protein-protein interaction protects NCM460 colonic cells and alleviates experimental colitis. *Sci. Rep.* **6**, 26585; doi: 10.1038/srep26585 (2016).

## Supplementary Material

Supplementary Information
