# Supplementary material for: An inhibitor of the Keap1-Nrf2 protein-protein interaction protects NCM460 colonic cells and alleviates experimental colitis
Source: Sci Rep. 2016 May 24;6:26585. doi: 10.1038/srep26585 (PMC4877580; doi:10.1038/srep26585)
Supplement: Supplementary Information [file srep26585-s1.doc]

Supporting Information

An inhibitor of the Keap1-Nrf2 protein-protein interaction

protects the NCM460 colonic cells and alleviates experimental colitis

Meng-Chen Lu,^a,b^ Jian-Ai Ji,^a,b^ Yong-Lin Jiang,^a,b^ Zhi-Yun Chen,^a,b^ Zhen-Wei Yuan,^a,b^ Qi-Dong You^a,b *^ and Zheng-Yu Jiang^a,b *^

^a^Jiang Su Key Laboratory of Drug Design and Optimization, China Pharmaceutical University, Nanjing 210009, China; ^b^Department of Medicinal Chemistry, School of Pharmacy, China Pharmaceutical Uni-versity, Nanjing 210009, China

S1. ITC (Isothermal Titration Calorimetry) Assay

S2. Cell Viability Assay

S3. Transfection of Small Interfering RNA (siRNA) against Nrf2 in Immunofluorescence Assay

S4. Transfection of a second Small Interfering RNA (siRNA) against Nrf2 in qRT-PCR Assay

S5. DSS-induced Pathogenic Conditions of Mice

S6. Statistical Analysis

**S1. ITC (Isothermal Titration Calorimetry) Assay**

The Isothermal titration calorimetry (ITC) was performed at 25 °C with the ITC200 system (MicroCal). The Keap1 Kelch domain was lyophilized. Both the protein sample and the conpound were dissolved in 10 mM HEPES buffer (pH 7.4). 2 μL aliquots of 0.05 mM compound were injected 19 times at 2.5 min intervals from a stirring syringe (750 rpm) into the sample cell containing 220 μl of 0.005 mM Keap1 Kelch domain. The data were analyzed with the computer program Origin, version 7.0, supplied by MicroCal.

**S2. Cell Viability Assay**

We examined the cytotoxicity of **CPUY192018** against the NCM460 cells using the MTT assay. Briefly, NCM460 cells in logarithmic phase were seeded at the density of 70 ~ 80% confluence per well in 96-well plates at 37°C with 5% CO_2_ for overnight incubation and treated with various concentrations (1.56 – 100 μM) of **CPUY192018** for 48 h. After treatment, 20 μl of 5 mg/ml MTT was added and the cells were incubated for 4 h at 37°C. The supernatant was discarded and 150 μl of DMSO was added to each well. The mixture was shaken on a mini shaker at room temperature for 5 min and the spectrophotometric absorbance was measured by Multiskan Spectrum Microplate Reader (Thermo, USA) at 570 nm and 630 nm. Triplicate experiments were performed in a parallel manner for each concentration point and the results were presented as the means ± SEM. The net A_570nm_-A_630nm_ was taken as the index of cell viability. The net absorbance from the wells of cells cultured with DMSO was taken as the 100% viability value. The percent viability of the treated cells was calculated by the formula: % viability = (A_570nm_-A_630nm_) _treated_/ (A_570nm_-A_630nm_) _control_×100%. The data in **Figure S1** showed that the survival rate remained higher than approximately 80% under 100 μM, indicating no apparent cytotoxicity for the treatment of **CPUY192018**.


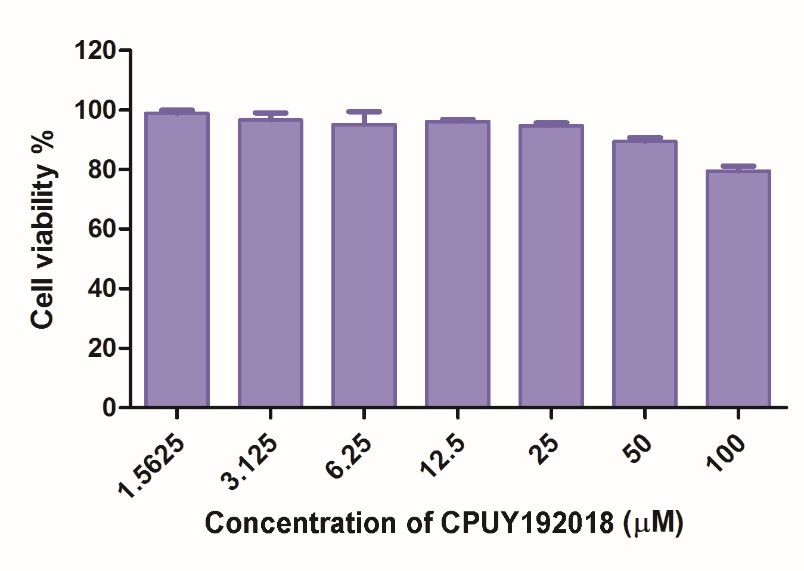


**Figure S1.** The cytotoxicity of **CPUY192018** against the NCM460 cells using the MTT assay.

**S3. Transfection of Small Interfering RNA (siRNA) against Nrf2 in Immunofluorescence Assay**

Predesigned siRNA against human Nrf2 (sc-37030) was purchased from Santa Cruz Biotechnology. NCM460 cells were plated at a density of 10×10^4^ cells in a six-well plate. Cells were transfected with 50 nM siRNA against Nrf2 using Lipofectamine 2000 (Invitrogen). After 24 h incubation, fresh medium was added, and the cells were cultured for another 48 h. The cells were then treated with CPUY192018 (10 μM) for indicated times, then incubated at 4°C overnight with Nrf2 primary antibodies (abcam, UK). After washing with PBS, cells were incubated at 37°C for 1 h with FITC-labeled secondary goat anti-rabbit IgG antibody (Life Technology). Cells were then stained with fluorochrome dye DAPI (Santa Cruz Biotechnology, Santa Cruz, CA) to visualize the nuclei and observed under a laser scanning confocal microscope (Olympus Fluoview FV1000, Japan) with a peak excitation wave length of 570 nm and 340 nm.


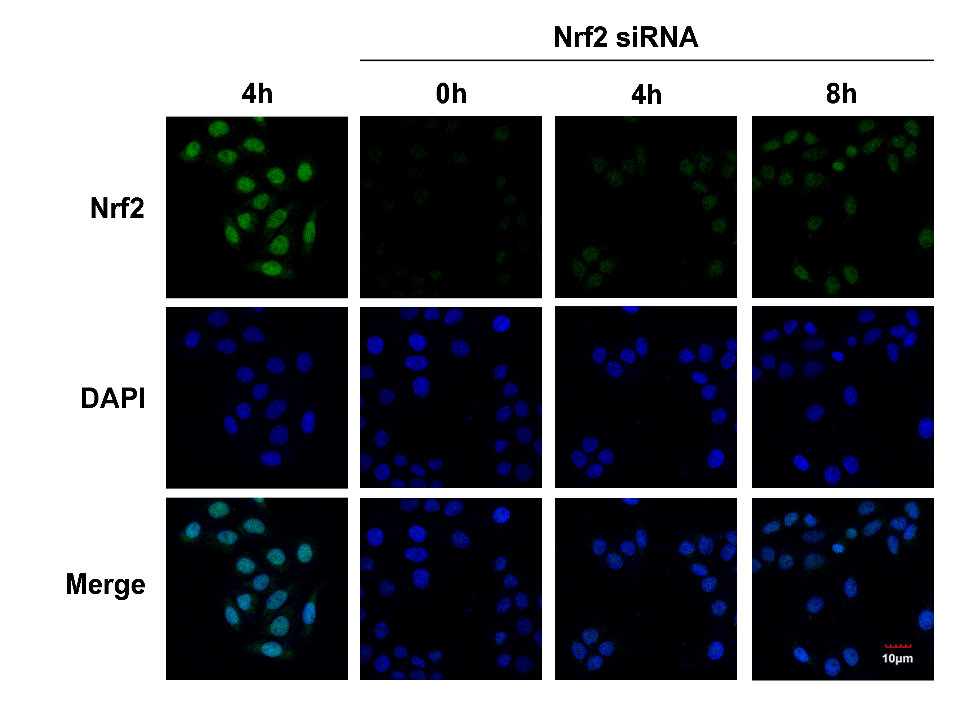


**Figure S2.** Nrf2 knockdown remarkably decreased the fluorescent intensity of Nrf2 in both nuclei and cytoplasm.

**S4. Transfection of a Second Small Interfering RNA (siRNA) against Nrf2**

Predesigned siRNA against human Nrf2 (sc-37030) was purchased from Santa Cruz Biotechnology. NCM460 cells were plated at a density of 6×10^5^ cells per 60 mm dish. Cells were transfected with 50 nM siRNA against Nrf2 or 50 nM scrambled duplex using Lipofectamine 2000 (Invitrogen). After 24 h incubation, fresh medium was added, and the cells were cultured for another 48 h. The cells were then treated with compounds for an additional 6 h and lysed for use in qRT-PCR.


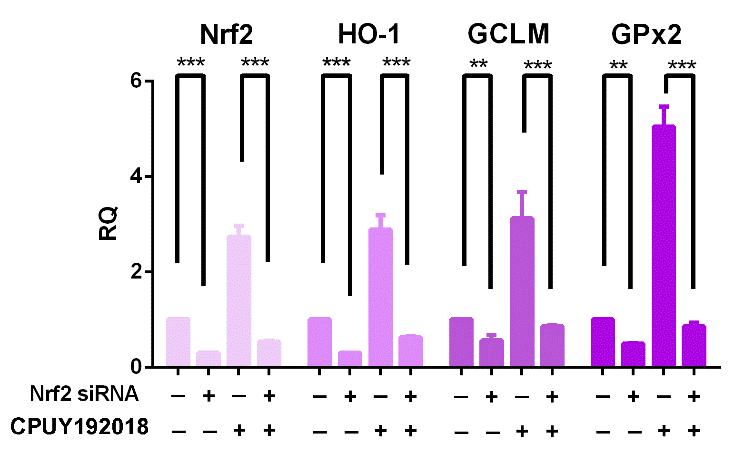


**Figure S3.** mRNA expression of Nrf2 and Nrf2-regulated genes after exposure to Nrf2 siRNA and **CPUY192018**. NCM460 cells were treated with Nrf2 siRNA (50 nM), **CPUY192018** (10 μM), or Nrf2 siRNA (50 nM) plus **CPUY192018** (10 μM). NCM460 cells treated with scrambled duplex were used as the blank control. The expression of Nrf2, HO-1, GCLM and GPx2 genes were quantified using qRT-PCR.

**S5. DSS-induced Pathogenic Conditions of Mice**

The DSS-induced pathogenic conditions were observed and recorded during the whole proces. Mice were checked by monitoring body weight every two days. Stool consistency (presence of diarrhea) and gross rectal bleeding (measured by visible fecal blood and macroscopic examination of the anus) were also recorded according to standard protocol. It was noted that there was a decrease in diarrhea and anus bleeding in mice treated **CPUY192018** coincident with DSS compared to those treated with DSS alone. Figure S4 showed that DSS-treated mice displayed shortened colon length and wide hemorrhages areas in colon (red arrowheads).

**Table S1. DSS-induced pathogenic conditions of mice.**

| Group | Body weight | Occult | Stool formation |
| --- | --- | --- | --- |
| Control | No change | negative | normal |
| DSS model | Decrease by 18.7% | positive, bleeding | very soft, diarrhea |
| DSS+CPUY192018(10 mg/kg) | Decrease by 13.4% | positive | soft but formed |
| DSS+CPUY192018(40 mg/kg) | Decrease by 8.8% | negative | normal |
| CPUY192018(40 mg/kg) | Decrease by 5.2% | negative | normal |


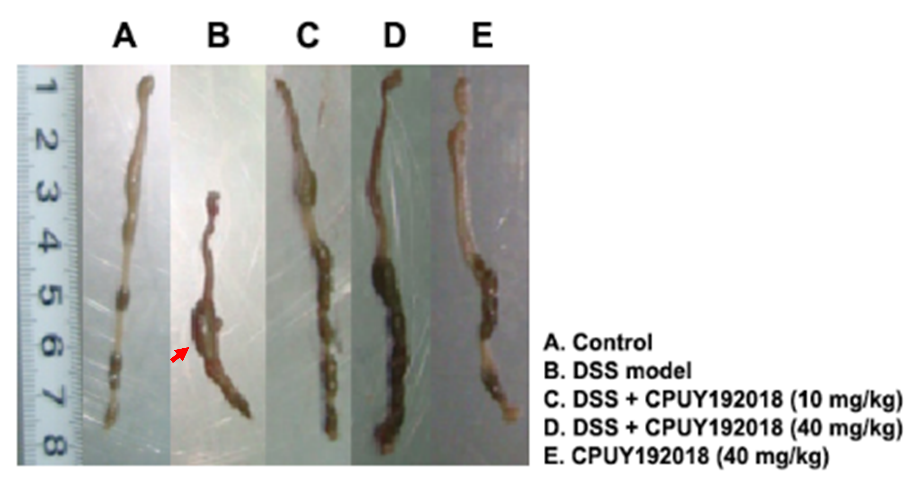


**Figure S4.** DSS-treated mice which displayed shortened colon length and wide hemorrhages areas in distal colon (red arrowheads).

**S6. Statistical Analysis**

Results are expressed as the means ± SEM. Statistical tests were performed using GraphPad Prism 6.0 software. For multiple comparisons between groups, a one-way ANOVA was performed to detect statistical differences. Differences within the ANOVA were determined using a Tukey’sposthoc test.
